# Dehydration Process of Hofmann-Type Layered Solids

**DOI:** 10.3390/ma6041452

**Published:** 2013-04-09

**Authors:** Omar Reyes-Martinez, Enelio Torres-García, Geonel Rodríguez-Gattorno, Edilso Reguera

**Affiliations:** 1 Centro de Investigación en Ciencia Aplicada y Tecnología Avanzada Unidad Legaria, Instituto Politécnico Nacional, Legaria 694, Colonia, Irrigación, México D.F. 11500, Mexico; E-Mails: oreyesm0621@hotmail.com (O.R.-M); edilso.reguera@gmail.com (E.R.); 2 Mexican Petroleum Institute, Eje Central 152, México D.F. 07730, Mexico; E-Mail: etorresg@imp.mx; 3 Center for Research and Advanced Studies of the National Polytechnic Institute, Km 6 Antigua Carretera Progreso, Cordemex, Mérida 97310, Yucatán, Mexico

**Keywords:** kinetics parameters, hi-res/modulated-TG, porous lattices, dehydration of lamellar solids

## Abstract

In the present work the dehydration process of layered solids with formula unit M(H_2_O)_2_[Ni(CN)_4_]·*n*H_2_O, M = Ni, Co, Mn; *n* = 1, 2, 4 is studied using modulated thermogravimetry. The results show that water molecules need to overcome an energetic barrier (activation energy between 63 and 500 kJ/mol) in order to diffuse through the interlayer region. The related kinetic parameters show a dependence on the water partial pressure. On the other hand, X-ray diffraction results provide evidence that the dehydration process is accompanied by framework collapse, limiting the structural reversibility, except for heating below 80 °C where the ordered structure remains. Removal of water molecules from the interlayer region disrupts the long-range structural order of the solid.

## 1. Introduction

For many years, microporous solids have played an important role in the improvement of technologies involving processes such as separation, adsorption, ion exchange and heterogeneous catalysis. For this reason, they have been extensively studied [1,2,3] and a new generation of different porous materials have emerged, such as metal–organic frameworks (MOFs) [4,5], covalent–organic frameworks (COFs) [6,7], zeolitic imidazolate frameworks (ZIFs) [8] and amorphous polymers of intrinsic microporosity (PIMs) [9], to mention just a few. There is another class of structures known as Hofmann-type compounds in which the size and geometry of the pores can be tailored by incorporating appropriate pillars between neighboring layers to form 3D porous frameworks. Such an assembling process impacts in the physico-chemical features, particularly in the geometry of the cavities and the surface properties for the resulting porous materials. Hofmann compounds are classified within the clathrate structures as microporous solids that have being the subject of intense research related to their fundamental significance in understanding the nature of interactions between molecular and ionic species [10,11,12,13]

The classical Hofmann structure consists of square planar Ni(II) metal centers surrounded by C-bound cyanide ligands, which are assembled by nickel atoms linked at the N end of the CN ligands [14]. In the resulting layer, the axial coordination positions for the second metal are occupied by NH_3_ or H_2_O molecules, which complete the metal octahedral coordination environment. The resulting motif is an extended 2D network in which octahedral nickel atoms can be replaced by several other transition metals to form solids with layered structures [15]. The region of weak interaction between neighboring layers, commonly known as the interlamellar region or gallery, is receiving increasing attention related to the possibility of utilizing the principles of crystal engineering and rational design to synthesize a great variety of inclusion compounds with predetermined structural characteristics. The series of Hofmann-type solids studied in this work are also appropriate building blocks for the formation of hybrid inorganic–organic materials, replacing the ammonia and water molecules coordinated to the axial positions of the octahedral metal by organic molecules [11,16]. When molecules with two coordination sites and different length are included in that region, pillared porous solids of different cavity size and geometry can be obtained [11]. When the organic ligand has only one coordination site, bimolecular pillars are formed [16]. In this last case neighboring layers remain in communication by the intermolecular interaction established between the organic ligands involved. In the interlayer region, the included molecules are confined to a small volume where their intermolecular interactions are limited to only few molecules.

These interesting compounds have potential applications in novel hybrid inorganic–organic materials and porous solids of tailored cavity geometry and volume appropriate for H_2_, N_2_ and CO_2_ storage and/or separation [10,11,12,13]. Hence, the aim of this work is to provide a detailed study of the thermal properties for a series of Hofmann-type layered solids in order to shed light on the structural stability of the water molecules upon heating and its related kinetic parameters. The kinetic parameters derived were used to interpret the mechanistic aspects related to dehydration and structural collapse of the solids considered in this work. The samples consist of solids of formula unit M(H_2_O)_2_[Ni(CN)_4_]·*n*H_2_O with M = Mn, Co, Ni, where neighboring layers remain together through a network of coordinated and hydrogen-bonded water molecules. The amount of weakly hydrogen-bonded water molecules found in the interlayers region determines the crystal structure adopted by the solids; three different ordered phases are known, L_0_, K and L_1_ [17,18,19], with 1, 2 and 4 weakly bonded water molecules per formula unit [19].

## 2. Experimental

### 2.1. Synthesis Procedure

**Figure 1:**
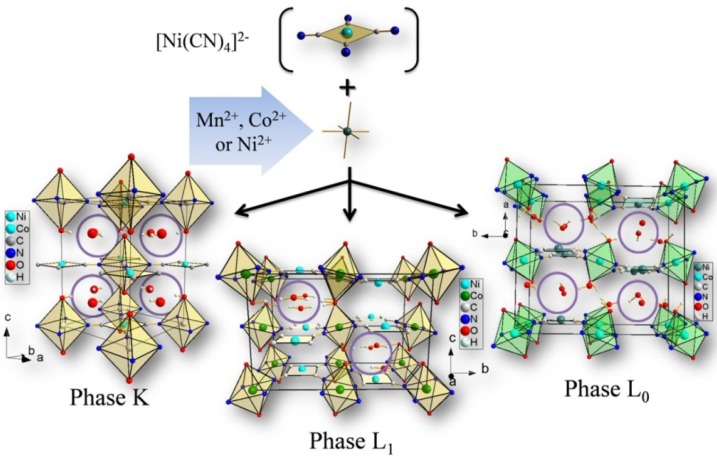
Schematic representation of the assembling process in order to obtain the K, L_1_ and L_0_ phases. The hydrogen-bonded water molecules are highlighted within open circles in the interlayers region, while the rest of the oxygen atoms (in red) correspond to coordinated water molecules (two per unit cell).

Samples were prepared by mixing aqueous solutions of 0.038 M K_2_[Ni(CN)_4_]·H_2_O and the corresponding metal sulfate (Mn^2+^, Co^2+^ and Ni^2+^) at the same concentration. For the preparation of the L_1_ phase, the solutions were mixed without agitation, while the L_0_ phase was precipitated with continuous stirring for 2 h. To obtain the K phase, the sample was maintained at 70 °C without agitation for 5 days. The resulting precipitated solids were separated from the solution by centrifugation. The solid fraction was repeatedly washed with distilled water to remove the accompanying ions and air-dried. The synthesis process is summarized in Figure 1, which illustrates the assembly of the anionic [Ni(CN)_4_]^2−^ building blocks with the M^2+^ metal cations to form a layered structure. The available water molecules used as solvent occupy the available axial positions for the assembling metal. These coordinated water molecules stabilize additional weakly bonded water molecules in the interlayer region through a network of hydrogen bonding interactions. As already mentioned, the amount of this type of water molecules determines the crystal structure of the formed precipitate.

### 2.2.X-ray Diffraction (XRD) and Scanning Electron Microscopy (SEM)

Powder X-ray diffraction patterns were collected for as-prepared samples and used to monitor the structural changes during the dehydration process. Patterns were obtained using Bragg-Brentano geometry in a D-8 Advance diffractometer with Cu Kα radiation, using a nickel filter in the secondary beam and a scintillation detector. Diffraction intensity was measured between 4° and 80°, with a step size of 0.02° and an integration time of 9 s per point. Details of the crystal structure for this family of 2D materials are available in reference [19], and the crystallographic information files were used to model the schematic structures of each phase and the water sub-networks. The morphology of the as-prepared and dehydrated samples was characterized by scanning electron microscopy (SEM) using a JEOL JSM 6390 LV operating at an accelerating voltage of 15~20 kV. Charging phenomena during observation did not allow us to obtain high quality images for some samples, while any attempt of increasing the conductivity by depositing a thin metallic gold layer by evaporation resulted in a change of the water content with partial structural collapse.

### 2.3.Hi-Resolution and Modulated Thermogravimetric Analysis

A Thermogravimetric Analyzer Q5000 IR by TA Instruments, Inc. (USA) was used to obtain mass loss profiles upon heating of the samples. Samples (between 5 and 10 mg) were placed in a platinum-HT open pan and heated from room temperature to 200 °C with a heating rate of 1 °C/min and a sinusoidal temperature perturbation with an amplitude of ±3 °C and a period of 60 s. These analyses were carried out both under a dynamic atmosphere of dry air and at a fixed water partial pressure (3130 Pa), with a flow of 25 cm^3^/min. The water partial pressure is controlled by bubbling the air through pure water at a constant temperature (25 °C). Typical thermogravimetric plots and the modulated parameters are shown in Figure S1 of the Supporting Information.

A slight difference between the experimental and theoretical weight loss was found, which was ascribed to a small fraction of physisorbed water molecules that are removed under gas flow during the programming of the MTG experiments. The resulting TGA curves were analyzed using “TA Instruments Universal Analysis 2000 software”. A detailed description related with the kinetic analysis can be found in section 1 of the Supporting Information.

It is important to note here that in order to get a clear comprehension of the general trends in the activation energy (*Ea*) profiles we should keep in mind the nature of the dehydration processes in each material under study (*i.e.*, hydrogen-bonded water molecules, coordinated water molecules or both). Assuming that it is reasonable to expect that the mechanism controlling the overall kinetics is similar for any specific crystallographic phase, we compare the activation energy profiles for each of the cations (*i.e.*, Ni^2+^, Co^2+^ and Mn^2+^). In general three regions can be distinguished along the dynamic activation energy profiles: high and steep sides at both extremes of the curve, and a nearly constant plateau in between, which can be considered as the minimum of the energy barrier (*Ea*) that should be overcome in the main process controlling the kinetics of the reaction [20]. Both extremes (at low and high *α* values) are zones where the mechanism is usually very complex due to its mixed nature; frequently these zones comprise simultaneous processes such as nucleation and growth, reaction and nucleation, or reaction and diffusion, therefore only the region for *Ea* from 0.1 ≤ *α* ≤ 0.9 is used for the interpretation of results. It should be noted that the activation energy is expected to depend on the transformation degree (*α*) [21,22,23]; in other cases, an increasing *Ea* as a function of *α* reveals a possible competition between parallel reactions [24,25,26,27].

## 3. Results and Discussion

### 3.1. Dehydration with Different Transition Metals in the Same Structure Phase

**Figure 2:**
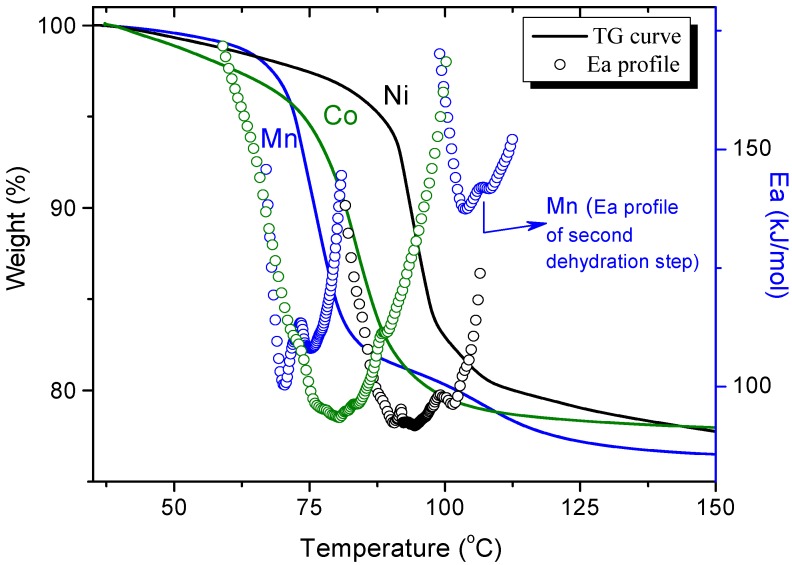
Thermogravimetric curves and activation energies profiles for the K-phase, M(H_2_O)_2_[Ni(CN)_4_]·2H_2_O, of Ni^2+^, Co^2+^ and Mn^2+^.

In Figure 2 the thermogravimetric curve (TG) and its activation energies profiles for Mn^2+^, Co^2+^ and Ni^2+^ K-phases are shown, which correspond to the case where the samples crystallized in the same phase (see X-ray diffraction patterns in Figure S2 of the Supporting Information) with a different transition metal in the structure. It can be observed that all samples result fully dehydrated above 180 °C. The temperature at maximum dehydration rate and the general material stability follow the order: Ni^2+^ > Co^2+^ > Mn^2+^, a sequence that is expected to be determined by the polarizing power of the metal and the strength of its electrostatic interaction with the water molecules included in its structural cavities [28]. In general, the O–O distance among water molecules in the framework always increases from Ni^2+^ to Mn^2+^ in each of the phases [19].

According to the activation energy profiles shown in Figure 2 for the K-phase of Co and Ni, the minimum values of the activation energy are quite similar. In addition, the dehydration process for the two metals occurs in a single step and without distinction for the release of hydrogen-bonded and coordinated water molecules. The mechanism for dehydration is envisaged to be controlled by the continuous local reordering of the water molecules induced by the water chemical potential at the solid-gas interface and along the structure. In the case of the Mn K-phase two dehydration steps are well resolved, the first one involving three (two hydrogen bonded and one coordinated) of the total o four water molecules, and the last step involving only one coordinated water molecule; here, the increase in activation energy is consistent with the energetic differences between the bonding nature expected for hydrogen-bonded and coordinated water molecules. For this metal (Mn), the activation energy corresponding to the second dehydration step increases up to 140 kJ/mol, a value that is typical for the removal of coordinated water molecules [28].

Following this reasoning, we assume that the release of water molecules with an energy barrier on the order of 90 to 100 kJ/mol corresponds to diffusional processes modulated by multiple hydrogen bonds, a behavior known for laminar systems or hydrated compounds [29,30,31,32].

### 3.2. Dehydration with the Same Transition Metals in Different Phases

**Figure 3:**
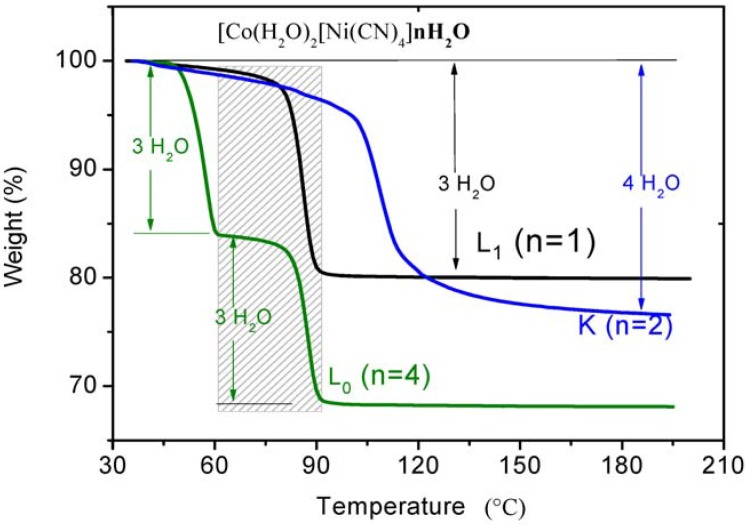
Thermogravimetric curves (TGs) for Co(H_2_O)_2_[Ni(CN)_4_]·*n*H_2_O, *n* = 1, 2, 4 (L_1_, K and L_0_ phases). The framed area emphasizes the similarity between the dehydration of the L_1_ sample (second step) and the L_0_ sample.

Figure 3 shows the TG curves for the same transition metal (Co^2+^) crystallizing in the 3 different structural modifications, wherein the total weight loss satisfactorily agrees with the number of water molecules found in each of the structures; 19.52% (2.98 water molecules), 23.42% (3.82 water molecules), and 31.88% (5.83 water molecules), which corresponds to the expected 3, 4 and 6 molecules for L_1_, K and L_0_ phases, respectively. In this case, the relative thermal stability order is L_0_ < L_1_ < K, which refers only to the completeness of the dehydration processes. Interestingly, in the L_0_ structure the shortest hydrogen bonds are found, hence, simple phenomenological arguments fail to explain the observed trend for the dehydration temperature. The interpretation of activation energy profiles in multistep dehydration processes of complex compounds is a challenging task related to the cooperative and anti-cooperative intermolecular interactions between water molecules and their host framework [33,34,35]. Another problematic issue in hydrogen bonding that is necessary to address for the interpretation of the dynamic rupture of hydrogen bonds is the local geometry of the water molecules network (homodromic, heterodromic or antidromic [36]); it is well known that the arrangement and clustering size affects the global energetics of the system [33].

**Figure 4:**
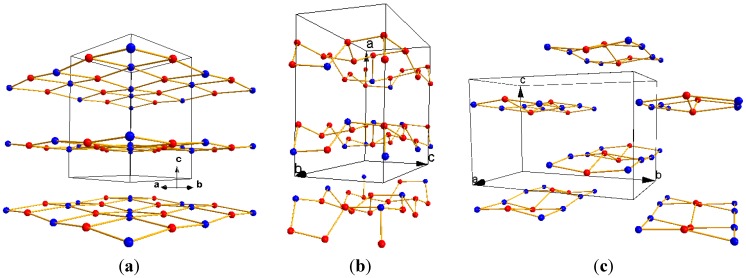
Schematic representation of water two-dimensional sub-network in (**a**) K; (**b**) L_0_; and (**c**) L_1_ phases. Each sphere represents oxygen atoms in water molecules: the atoms in blue correspond to coordinated water molecules (note that the hydrogen atoms are omitted). The black lines delimit the unit cell in each structure and the arrows indicate the unit cell axes.

In the present Hofmann’s crystal structures the water molecules organize along two-dimensional planes (see schematic representation in Figure 4), alternating their positions between the fixed octahedral coordination sites (defined by the metal position) and positions within the cavities in between the metals polyhedrons. Note that the water network consists of a continuous two-dimensional tetramer arrangement for the K phase, a hexagonal ice-like, two-dimensional network for the L_0_ phase and discrete arrangements of tetramers for the L_1_ phase. According to theoretical quantum calculations the tetramer configuration appears to represent the most stable water cluster [33], while the shortest hydrogen bonds of the L_0_ phase, which have a hexagonal ice-like configuration, are not necessarily the strongest [37].

Following Figure 3, the phases L_1_ and K release the water molecules in a single step, indicating that the dehydration process takes place through a dynamic interchange between the water molecules located at diffusion channels and those at coordination positions in both structures. The first step in the TG curve, for the L_0_ phase, involves the loss of only three of the total four water molecules that are weakly interacting with the structure through hydrogen bonds; this takes place at a relatively low temperature (60 °C, see Figure 3). Subsequently, the coordinated water molecules of the L_0_ phase are released at 90 °C in a profile which resembles that of the L_1_ phase (see the framed area in Figure 3). This behavior is quite interesting because it suggests the occurrence of the transition from the L_0_ to the L_1_ phase after the release of the water molecules interacting through hydrogen bonds.

**Figure 5:**
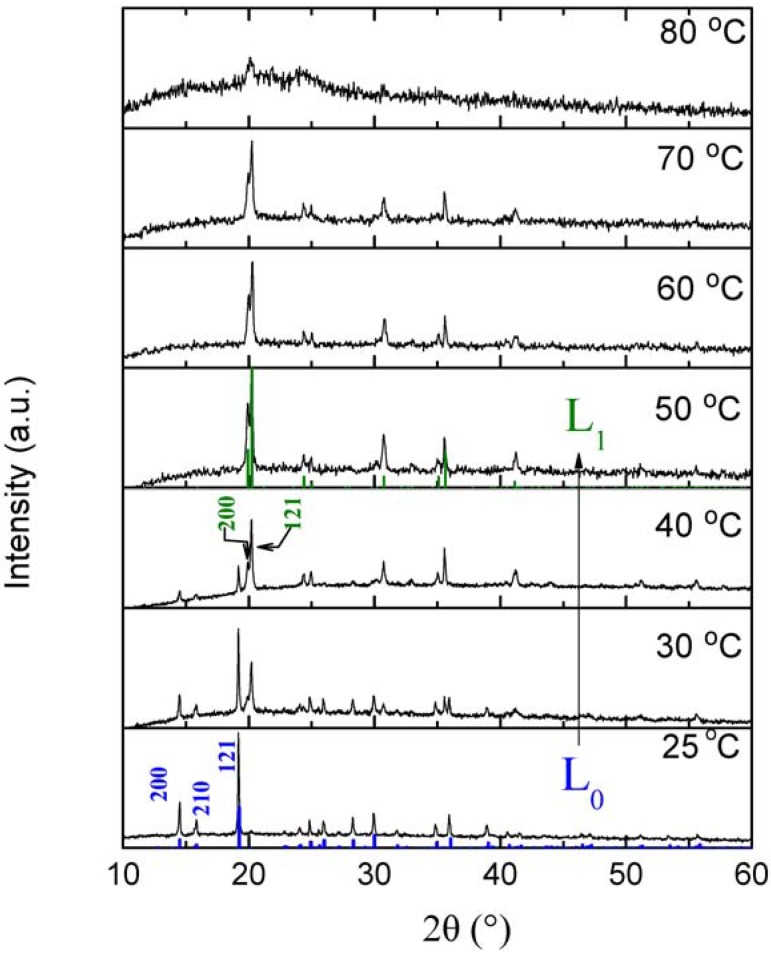
Sequence of X-ray powder diffraction patterns of as-prepared Co(H_2_O)_2_[Ni(CN)_4_]·4H_2_O (L_0_ phase) from room temperature to 80 °C, showing the transition to the L_1_ phase upon dehydration and its further collapse into an amorphous material. Colored bar-plots correspond to diffraction lines expected for each of the pure phases: L_0_ (blue) and L_1_ (olive).

In order to corroborate the possible L_0_ to L_1_ structural transition, the L_0_-[Co(H_2_O)_2_Ni(CN)_4_]·4H_2_O sample was heated from room temperature to 80 °C and monitored every 10 °C by X-ray diffraction. Figure 5 shows the sequence of the X-ray diffraction patterns upon heating, in which the phase transition and finally the structural collapse once the dehydration completes at 80 °C can be clearly observed. According to the peak positions, the transition and collapse occur gradually but without noticeable change of the cell parameters.

The transition involves a reduction of 25.3% of the cell volume, mainly resulting from a considerable contraction in the *a* axis parameter as shown in Table 1, which is also accompanied by a less pronounced expansion in the *b* and *c* axes. From Figure 5, the most noticeable changes are the collapse of the L_0_-{200}, {210} and {121} families of planes and their reconstruction in {002} and {121} of the L_1_ phase. As expected from the similarities between the structures, the crystallographic transition takes place in all the L_0_ phases of each metal, in a similar range of temperatures (see Figure S3 in Supporting Information). It can be envisaged that the loss of the shielding effect from water molecules upon dehydration of the L_0_ phase generates a local charge imbalance that provokes the displacement of sheets toward better stability (L_1_ phase). According to the temperature dependence of the cell parameters, this process seems to be critical rather than gradual; also note that both phases could coexist even at temperatures as low as 40 °C. In the L_0_ phases the only step worthwhile to analyze is the first dehydration step related to the L_0_ transition into the L_1_ phase; the stability follows the expected trend according to the O–O distance, however, the activation energy for Ni^2+^ and Mn^2+^ are essentially the same (108 and 106 kJ/mol, respectively) while for Co^2+^ it increases to 168 kJ/mol. There is no simple, straightforward explanation for this tendency, however, if one analyzes the temperatures of the maximum rate of dehydration, the order is Co^2+^ (46 °C), Mn^2+^ (52 °C) and Ni^2+^ (58 °C); hence, the Co–L_0_ phase is more affected by the simultaneous presence of both phases and in the mixed character of the mechanism.

**Table 1:** Calculated cell parameters and volume for L_0_ and L_1_ phases.

	L_0_ phase	L_1_ phase
Cell Parameters (Å)	a = 12.195(2)	a = 7.115(2)
	b = 13.885(3)	b = 14.264(3)
	c = 7.143(3)	c = 8.898(1)
Volume (Å^3^)	1209.5(3)	903.0(2)

**Figure 6:**
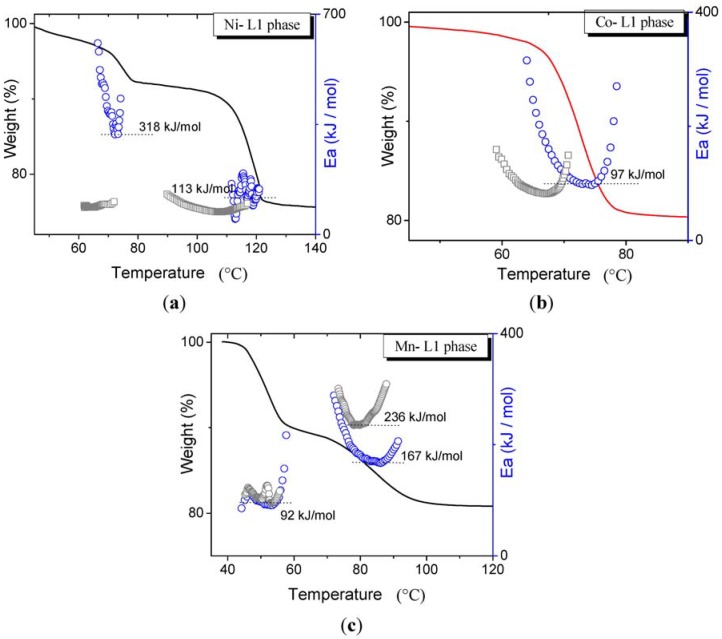
Thermogravimetric curves and the activation energy profiles for the three L_1_ phases (M(H_2_O)_2_[Ni(CN)_4_]·2H_2_O, where M = (**a**) Ni^2+^; (**b**) Co^2+^ and (**c**) Mn^2+^). Gray points in each of the plots correspond to activation energy profiles of the L_1_ phase resulting from heating of the L_0_ phase.

On the other hand, the L_1_ phases involve two main steps of dehydration except for Co, where dehydration occurs in only one step; in Figure 6 the thermogravimetric curves with their corresponding activation energy profiles for each of the metals are shown.

**Figure 7:**
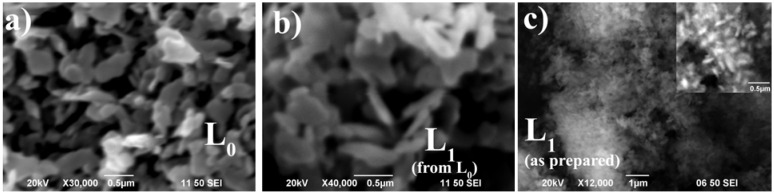
SEM micrographs of: (**a**) as prepared L_0_ phase; (**b**) L_1_ derived from L_0_ after heating and (**c**) the as prepared L_1_ phase, the insets shows a magnification of a selected area.

Here it is worth mentioning that as L_0_ transforms into L_1_ during dehydration, we would expect that *Ea* for the second step of dehydration of L_0_ were similar to those measured in pure as-prepared L_1_. This inference is essentially true, within 10% of the minimum energy value, except for nickel in which the value increased to more than threefold in the first step, while for manganese the activation energy decreased from 236 to 167 kJ/mol in the second step (see gray points in the plots). Also observe that the general tendency is that L_1_ derived from L_0_ (during thermogravimetric studies) always have a slightly lower dehydration temperature. The calculated cell parameters and the weight loss for both types of L_1_ phases (as prepared or obtained from thermogravimetric study) are the same, therefore, changes in activation energies cannot be attributed to structural dissimilarities or to differences in the composition and content of water in the structure. As the changes in morphologies might strongly influence the diffusional paths, additional SEM characterization were performed in order to explain this behavior (see, for example, Figure 7).

**Figure 8:**
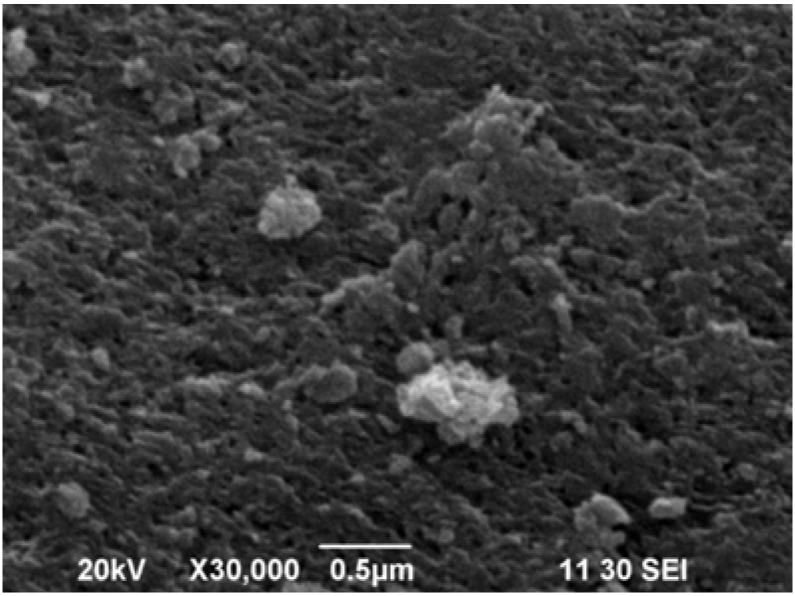
SEM micrograph of the as prepared Ni-L_0_ phase.

As can be observed in Figure 7a,b, the structural transition takes place without change in the morphology of the crystallites in spite of the volume change during transformation. Hence, the L_1_ phase derived from L_0_ conserves the initial L_0_ morphology. In general, all samples show a plate-like morphology which is a manifestation of the lamellar character of the crystal structure; however, substantial differences are found in crystal cleavage and flatness of the microcrystals. Contradictorily, the as-prepared L_1_ phase exhibits a smaller particle size than L_1_ derived from heated L_0_, suggesting that lowering in dehydration temperature is not directly related to the diffusional pathway during water release. Despite the low quality of the micrograph of the Ni-L_0_ phase, it appears that this sample is different from the rest of the L_0_ materials by having very small and organized flake-like crystals (see Figure 8) with an interparticle mesoporous morphology, which could explain the lower activation energy for Ni-L_1_ derived from L_0_.

Samples of anhydrous solids obtained by progressive and moderate heating on the herein studied materials for the three considered metals have been used in other work for pyrazine and imidazole intercalation, and 3D crystalline solids were always formed [10,11,12,13,38,39]. This is in correspondence with the recorded TG curves. Once all the water molecules are removed, the layered material remains stable and only above 280 °C it is progressively disrupted with the evolution of cyanogen, C_2_N_2_, and the formation of reduced species of the involved metals, which are then oxidized in the presence of air.

### 3.3. The Influence of Water Partial Pressure

**Figure 9:**
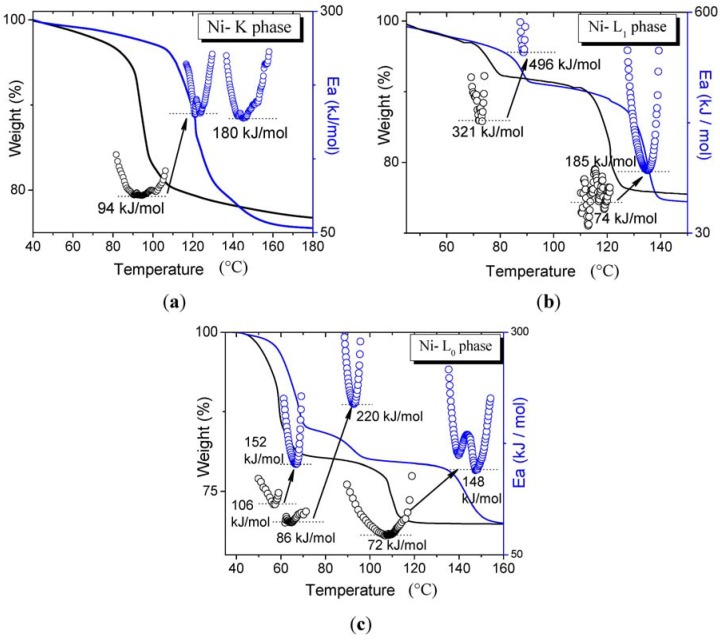
Thermogravimetric curves and their corresponding *Ea* profiles for (**a**) Ni-K phase; (**b**) Ni-L_1_ phase and (**c**) Ni-L_0_ phase. The data in blue corresponds to samples under higher water partial pressure (3130 Pa), while plots in black represent the profiles without water. Arrows indicate the correspondence between each step, with and without the presence of water.

With the purpose of observing the influence of chemical potential of water in the kinetics of the dehydration processes, the thermal profiles were studied under fixed water partial pressure (3130 Pa) in the purging gas flow, and some of the results are presented in Figure 9 as an example. For comparison purposes, thermogravimetric curves without the presence of water are also shown (plots in black).

It can be observed that there is a shift of all the curves to higher temperatures due to the increase of the water partial pressure; this is a general tendency for all the samples. The shift in the temperature corresponding with the maximum rate ranges from 10 °C to 35 °C and is accompanied by a substantial increase of the minimum activation energy. The temperature increase is intuitively expected on the basis of the Le Chatelier’s principle; however, the observed increase in activation energy implies a considerable reduction of the dehydration rate, which is an indication of a transition state characterized by a dissociative mechanism (negative activation volume change, see details in Section 2 of the Supporting Information). This is also predictable behavior since dehydration processes mainly involve the breaking of hydrogen and coordination bonds. In general, it is noticed that increasing the water partial pressure increases the *Ea* dependence on the reaction advance, hence, the activation energy profiles become more pronounced and symmetric around a minimum close to *α* ≅ 0.5, which is an indication of the complex nature of the dehydration process [29,40]. In some cases (Ni-K phase, Figure 9a), the *Ea* profile splits into two well-resolved processes with similar values of the activation energy, suggesting a separation of contributions from hydrogen bond water and the coordinated water molecules, and supporting the idea that both types of water molecule leave the material by the same mechanism. However, although the results are reproducible, further detailed research is necessary to discard the possible influence of experimental parameters. In the presence of higher water partial pressure, all the previous trends change, where the K phase appears to be more affected than any other phase; for example, the Co-K phase suffers an increment of the activation of more than three times the initial value.

The Ni-L_1_ phase is the only case where the activation energies result to be surprisingly high in the first step of dehydration (even without the presence of water). There are few examples in the literature that report similar values for dehydration processes [40,41,42]; we assume that this behavior could be related to the discrete character of the water sub-network in this phase which, in turn, increases the initial barrier for water diffusion; however, further exhaustive research is required for a better insight into this phenomenon.

## 4. Conclusions

The dehydration and structural collapse of the Hofmann’s layered compounds, M(H_2_O)_2_[Ni(CN)_4_]·nH_2_O with M = Mn^2+^, Co^2+^, Ni^2+^ and n = 1, 2 4 were studied by means of modulated thermogravimetry and X-ray diffraction techniques. In general, the temperature at the maximum dehydration rate and the material stability follows the order: Ni^2+^ > Co^2+^ > Mn^2+^, a sequence that is determined by the metal polarizing power and the strength of its electrostatic interaction with the water molecule included in its structural cavities, and defines the mean intermolecular distance. Themogravimetric curves and X-ray diffraction patterns show that during the first step of dehydration L_0_ transforms into the L_1_ phase, and the subsequent dehydration at higher temperatures entails the structural collapse of the materials; the phase transition results from the loss of the shielding effect from water molecules upon dehydration, which generates a local charge imbalance provoking the displacement of sheets toward better stability (L_1_ phase). The increase in water partial pressure results in a shift of the thermogravimetric curves to higher temperatures, accompanied by an increase of the activation energy in most of the materials. The dehydration process takes place without disruption of the layer structure. Only above 280 °C, is progressive decomposition of layers upon heating observed.

## Supplementary Materials

Supplementary File 1
