# Supplementary material for: Dehydration Process of Hofmann-Type Layered Solids
Source: Materials (Basel). 2013 Apr 9;6(4):1452–66. doi: 10.3390/ma6041452 (PMC5452313; doi:10.3390/ma6041452)
Supplement: Supplementary File 1 [file materials-06-01452-s001.pdf]

Supporting Information

S1. Kinetic Analysis under Modulated Conditions

This section describes basic details related to the application of modulated thermogravimetry to the kinetic study accomplished in the present work. Thermogravimetry is a fast tool for obtaining information about the kinetics of thermal degradation processes; the temperature dependence of chemical processes may be readily expressed in terms of the classical Arrhenius equation. In a modulated heating rate method, a sinusoidal temperature perturbation is superimposed on the conventional linear heating profile according to:

$$T_m(t) = T_0 + at + A\sin(2\omega t) \quad (1)$$

First two terms are related to the linear time-temperature dependence in classical thermogravimetry ($T_m = T_0 + at$), where T_0 is the initial temperature (room temperature), t is time, a is the heating rate (dT/dt = constant), ω is a frequency and A is the amplitude of the modulation [1]. Following the Arrhenius formalism, the basic equation that defines the rate of a heterogeneous reaction can be expressed as [2]:

$$\frac{d\alpha}{dt} = f(\alpha)\, Z\; e^{\left(-\frac{Ea}{RT}\right)} \quad (2)$$

where Ea is activation energy of the elemental process that determines the overall kinetics, Z is the Arrhenius pre-exponential factor, α represents the reaction progress as a solid fraction which has reacted after time t, and $f(\alpha)$ is a function related to the mechanism that controls the reaction rate. Equation (2) may be evaluated in a sine wave modulated experiment as the ratio of the periodic rate of reaction in adjacent peaks, and valleys [3], hence:

$$\frac{(d\alpha_p/dt) = f(\alpha)_p\, Z\; e^{\left(-\frac{Ea}{RT_p}\right)}}{(d\alpha_v/dt) = f(\alpha)_v\, Z\; e^{\left(-\frac{Ea}{RT_v}\right)}} \quad (3)$$

where, v and p sub-indices denote the rate, the temperature and the $f(\alpha)$ at the peak and valley. For small changes in temperature the reacted fraction changes little between adjacent half cycles of the modulated signal, in which case the value for $f(\alpha)_p$ approaches that of $f(\alpha)_v$ and their ratio approaches unity. Solving for Ea and rearranging gives:

$$Ea = \frac{RT_pT_v \ln(d\alpha_p / d\alpha_v)}{T_p - T_v} \quad (4)$$

Note that, T_p and T_v can be expressed as $T + A$ and $T - A$ while $T_p - T_v$ can be substituted by 2A which reduces Equation (4) to:

$$Ea = \frac{R(A^2 - T^2)\ln(d\alpha_p / d\alpha_v)}{2A} \quad (5)$$

Now we have an equation where the perturbation of the derivative of mass conversion is proportional to activation energy disregarding the nature of the mechanism. The rate of weight loss

responds to the temperature oscillations and the use of discrete Fourier´s transform allows one to calculate the kinetic parameters *Ea* and *Z* on a continuous basis [4–6]. This enables the study of the decomposition kinetics as a function of time without any assumption about the reaction mechanism (model-free calculations). The approach may be used under quasi-isothermal conditions to observe a single weight loss or may be combined with linear or nonlinear heating (*i.e.*, dynamic high-resolution thermogravimetry) to scan from one weight loss region to another. This approach provides continuous kinetic information in a fraction of the time required for traditional TGA kinetic experiments. Typical modulated thermogravimetry curves and its related parameters are shown in Figure S1.

Figure S1. Typical modulated thermogravimetric curves showing the modulated temperature, weight loss, modulated derivate of weight and the activation energy profile. The example corresponds to the Co-L_0 phase.

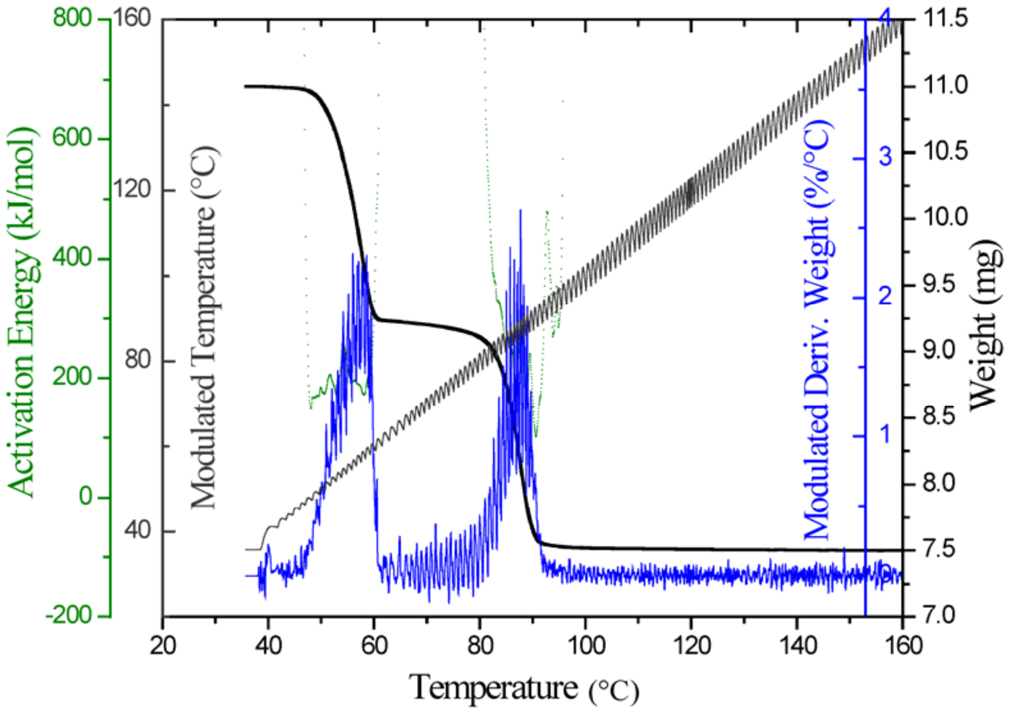

S2. Dependence of Reaction Rate with Water Partial Pressure

Studies of the pressure dependence of the reaction rate can give very useful information on the rate-determining step and on the reaction mechanism. According to transition state theory, the determination of the rate constant as a function of temperature at constant pressure provides the activation energy according to:

$$(\frac{\partial \ln k}{\partial T})_P = -\frac{Ea}{RT} \tag{6}$$

while the activation volume $\Delta V^{\neq}$, (*i.e.*, the volume difference between the transition state complex and the reactants) as a function of pressure at constant temperature is expressed as [7]:

$$(\frac{\partial \ln k}{\partial P})_T = -\frac{\Delta V^{\neq}}{RT} \tag{7}$$

or, in integrate form:

$$\ln\left(\frac{k_{(P_1)}}{k_{(P_2)}}\right) = -\frac{\Delta V^{\neq}}{RT}(P_1 - P_2) \tag{8}$$

Here, P_1 and P_2 refer to the equilibrium pressure and the actual pressure, respectively, and their rate constants at each pressure ($k_{(P1)}$ and $k_{(P2)}$). Reactions involving an associative transition state, where the rate-determining step involves the formation of a bond, give rise to a negative $\Delta V^{\neq}$, while dissociative type reactions involving the breaking of a bond present a positive $\Delta V^{\neq}$.

Figure S2. Experimental X-ray diffraction patterns of K-M[Ni(CN)$_4$] phases (M = Ni, Mn and Co). The blue lines correspond to the expected reflections for each sample calculated from crystallographic information files.

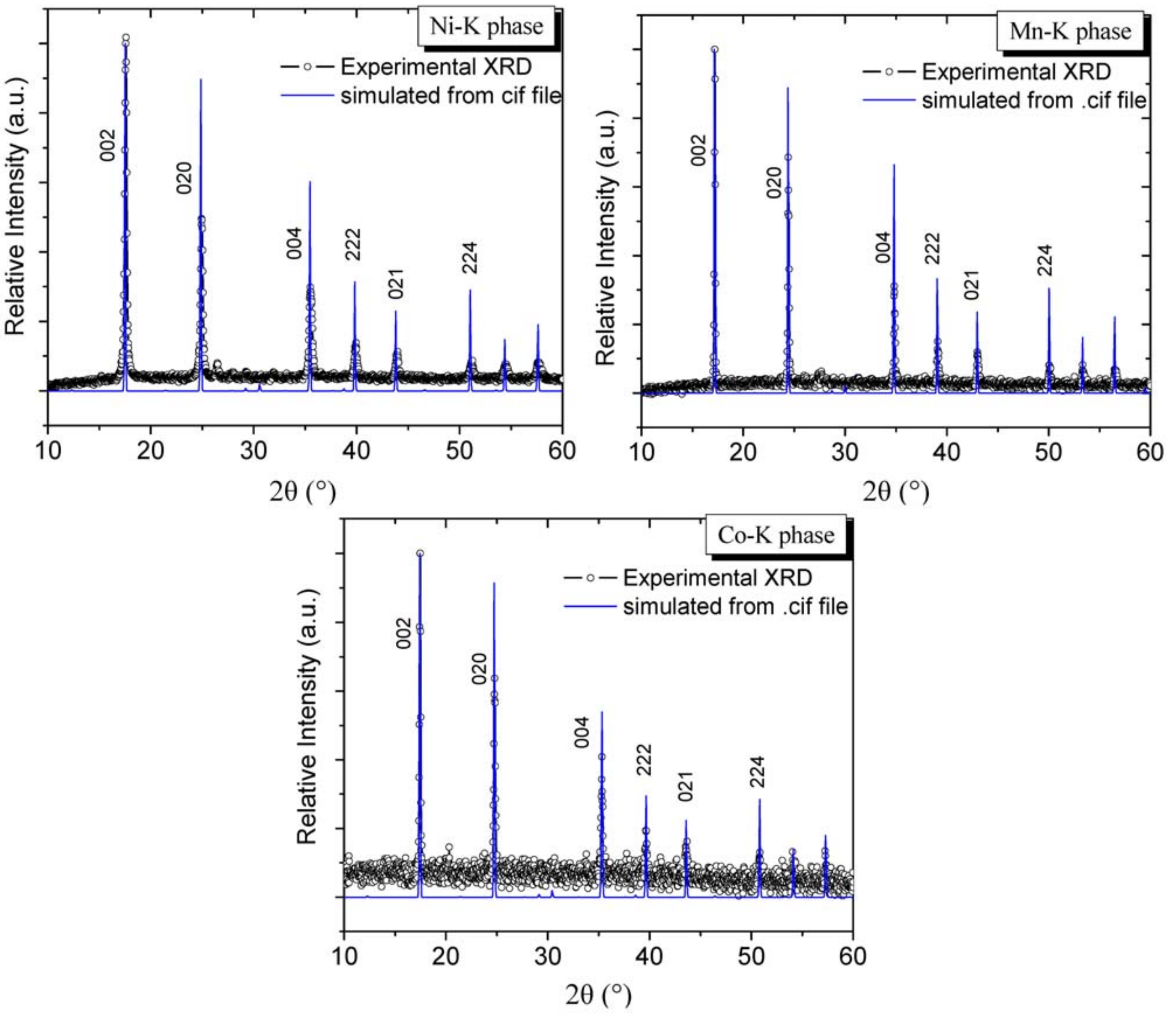

Figure S3. X-ray diffraction patterns showing the transition from L_0 to L_1 during L_0 phase dehydration, which corresponds to the process: $M(H_2O)_2[Ni(CN)_4]\cdot 4H_2O \rightarrow M(H_2O)_2[Ni(CN)_4]\cdot H_2O + 3H_2O$; where M = Ni^{2+} (left plot) and Mn^{2+} (right plot). Colored bar plots correspond with diffraction lines expected for each of the pure phases: L_0 (blue) and L_1 (olive).

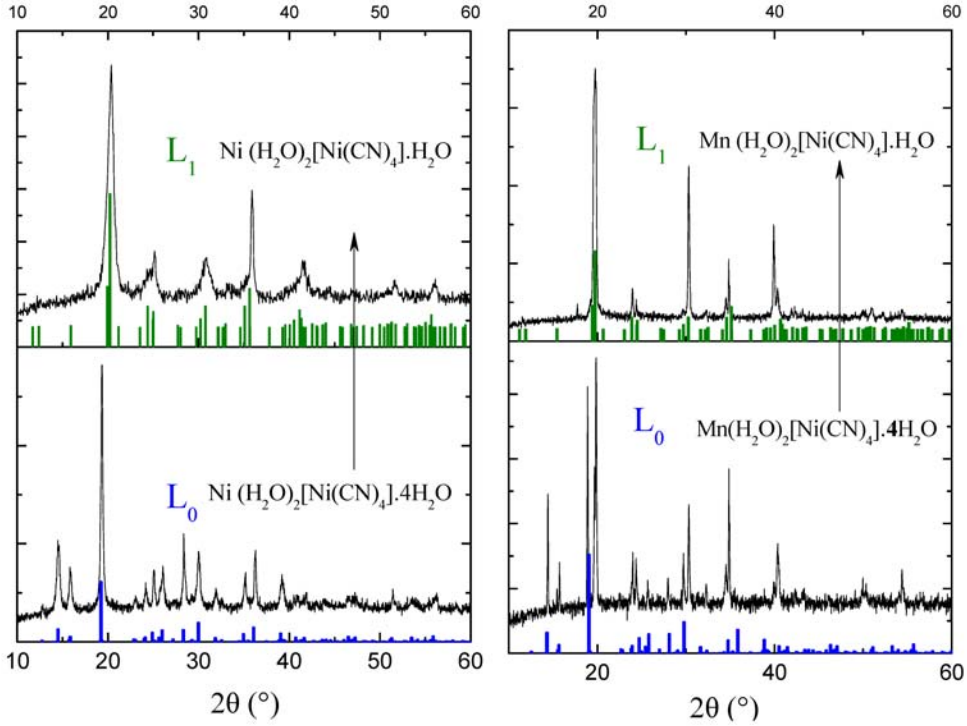

References

1. Mamlev, V.; Bourbigot, S. Calculation of activation energies using the sinusoidally modulated temperature. *J. Therm. Anal. Calorim*. **2002**, *70*, 565–579.
2. Vyazovkin, S.; Wight, C.A. Kinetics in solids. *Annu. Rev. Phys. Chem.* **1997**, *48*, 125–149.
3. Flynn, J.H.; Dickens, B. Steady-state parameter-jump methods and relaxation methods in thermogravimetry. *Thermochim. Acta* **1976**, *15*, 1–16.
4. Keuleers, R.R.; Janssens, J.F.; Desseyn, H.O. Comparison of some methods for activation energy determination of thermal decomposition reactions by thermogravimetry. *Thermochim. Acta* **2002**, *385*, 127–142.
5. Reading, M.; Kahn, B.K.; Crow, B.S. Method and Apparatus for Modulated Differential Analysis. *US Patent 5,224,775*, 6 July 1993.
6. Galwey, A.K. Structure and order in thermal dehydrations of crystalline solids. *Thermochim. Acta* **2000**, *355*, 181–238.
7. Evans, G.; Polanyi, M. Some applications of the transition state method to the calculation of reaction velocities, especially in solution. *Trans. Faraday Soc.* **1935**, *31*, 875–894.

© 2013 by the authors; licensee MDPI, Basel, Switzerland. This article is an open access article distributed under the terms and conditions of the Creative Commons Attribution license (http://creativecommons.org/licenses/by/3.0/).
